# Analysis of Registered Clinical Trials in Surgical Oncology, 2008-2020

**DOI:** 10.1001/jamanetworkopen.2021.45511

**Published:** 2022-01-27

**Authors:** Bonnie O. Wong, Nirosha D. Perera, Jolie Z. Shen, Brandon E. Turner, Henry K. Litt, Amit Mahipal, Sherry M. Wren

**Affiliations:** 1 Stanford School of Medicine, Stanford, California; 2 Department of Medicine, Mayo Clinic, Rochester, Minnesota; 3 University of Washington School of Medicine, Seattle; 4 Department of Radiation Oncology, Harvard Medical School, Boston, Massachusetts; 5 Department of Medicine, University of California, San Francisco; 6 Department of Oncology, Mayo Clinic, Rochester, Minnesota; 7 Department of Surgery, Stanford University, Stanford, California; 8 Palo Alto Veterans Health Care System, Palo Alto, California

## Abstract

This quality improvement study characterizes surgical oncology trials, analyzes growth, identifies associations with early discontinuation or results reporting, and evaluates proportions of trials involving each neoplasm site.

## Introduction

Surgical interventions are essential in the treatment of solid organ tumors, yet surgical interventions are infrequently studied via clinical trials.^1,2^ This study characterizes surgical oncology trials registered on ClinicalTrials.gov, analyzes growth, identifies associations with early discontinuation or results reporting, and evaluates proportions of trials involving each neoplasm site.

## Methods

This qualitative improvement study was reviewed by the institutional review board at Stanford University and was exempt from oversight and informed consent because all data are publicly available. Our findings are reported in concordance with the Strengthening the Reporting of Observational Studies in Epidemiology (STROBE) reporting guideline.

In this study, we downloaded 270 172 studies that were registered on the Aggregate Analysis of the ClinicalTrials.gov database from October 1, 2008, to March 9, 2020. Noninterventional trials were excluded, and oncology-specific Medical Subject Heading terms were used with a published protocol.^3,4^ In total, 27 915 trials were manually reviewed by the Clinical Trials Research Team to determine oncology relevance, intervention studied, and neoplasm site (Table). Surgical trials indicate surgical intervention as a study variable. Primary exposure variables were trial focus (eg, intervention, neoplasm site) and funding (eg, industry, US government, academic). Primary outcomes were early discontinuation and results reporting in the ClinicalTrials.gov database. Missing data were addressed via multiple imputations via a published protocol^4^

**Table.** Characteristics of Surgical Oncology Trials Compared With All Other Oncology Clinical Trials From 2008-2020

Characteristics	No. (%)	
	Surgical oncology trials (n = 1646)	All other oncology clinical trials (n = 20 433
Primary purpose		
Treatment	1645 (99.9)	14 607 (71.5)
Prevention	0 (0)	264 (1.3)
Basic science, other, or missing	1 (0)	5562 (27.2)
Funding		
Industry	177 (10.8)	6905 (33.8)
Academic	1368 (83.1)	11 191 (54.8)
US government	101 (6.1)	2337 (11.4)
Trial enrollment, No. of patients		
0-9	142 (8.6)	1911 (9.4)
10-49	446 (27.1)	6771 (33.1)
50-99	311 (18.9)	4099 (20.1)
100-499	609 (37.0)	5930 (29.0)
500-999	89 (5.4)	979 (4.8)
≥1000	48 (2.9)	710 (3.5)
Randomization		
Nonrandomized	677 (41.1)	10 869 (53.2)
Randomized	961 (58.4)	9158 (44.8)
Missing	8 (0.1)	406 (2.0)
Blinding		
None	1261 (76.6)	16 825 (82.3)
Double	92 (5.6)	1966 (9.6)
Single	288 (17.5)	1529 (7.5)
Missing	5 (0.3)	113 (0.6)
Sites, No.		
1	1128 (68.5)	11 025 (54.0)
2	96 (5.8)	1389 (6.8)
3-10	165 (10.0)	2813 (13.8)
>10	121 (7.4)	3659 (17.9)
Missing	136 (8.3)	1547 (7.6)
Study groups		
1	574 (34.8)	8865 (43.4)
2	961 (58.4)	8758 (42.9)
≥3	89 (5.4%)	2245 (11.0)
Missing	22 1.3)	565 (2.8)
Early trial discontinuation	181 (11.0)	2656 (13.0)
Results reported	127 (7.7)	3078 (15.1)

Solid organ tumor clinical trials were manually reviewed by the Clinical Trials Research Team to determine intervention focus and each trial was assigned to 1 or multiple appropriate categories of interventions, including (1) radiation, (2) surgical, (3) other invasive (nonsurgical and nonradiation, eg, cryoablation, thermoablation, endoscopic stent placement), (4) medical, and (5) other (eg, physical therapy, counseling). Clinical trials comparing 2 intervention types in separate groups were assigned both intervention types. Clinical trials comparing 2 or more intervention types were assigned only the intervention type that varied between separate groups. Clinical trials examining multiple interventions in a single group were assigned for all interventions involved.

Each labeler was provided a set of rules and examples to manually categorize clinical trials. Each labeler first achieved more than 90% agreement in categorizing a training set of trials (set by B. E. T.). A subset of each labeler’s categorization was reviewed by another labeler to ensure agreement.

We assessed descriptive statistics using 2-sided Pearson χ^2^ tests. We assessed trends over time using compound annual growth rates and Mann-Kendall significance tests. All analyses were 2-sided, and statistical significance was set at *P* = .05. Statistical analyses were performed between April 2020 and November 2021. All data were analyzed using R version 3.5.2 (R Project for Statistical Computing).

## Results

In this study, 26 815 of 27 915 trials had an oncology focus, of which 22 079 focused on solid organ tumors, and 1646 (7.6%) involved surgical intervention. Surgical oncology trials increased at a rate of 9.5% from 2008 to 2020 (compared with a compound annual growth rate of 5.0% for oncology trials overall). The growth rates of surgical oncology trials originating from North America (2.3%), Europe (13.9%), and East Asia (11%) were outstripped by the growth rate from countries outside of these 3 regions (25.1%).

Of surgical oncology trials, 961 (58.5%) were randomized, 1261 (76.6%) were not blinded, 1128 (68.5%) were from a single-institution, and 961 (58.4%) had 2 groups. A higher proportion of surgical trials received academic funding compared with all other oncology trials (1368 [83.1%] vs 11 191 [54.8%]) (Table) and a lower proportion reported results (127 [7.7%] vs 3078 [15.1%]). Among surgical trials, US government–funded trials had the lowest risk of early discontinuation (0.50; 95% CI, 0.26-0.99; *P* = .05) and academic-funded trials had lower odds of results reporting (0.29; 95% CI, 0.17-0.49 CI; *P* < .001) using industry-funded trials as a reference.

The most studied neoplasia sites for surgical oncology trials were colorectal (175 [17.6%]), breast (290 [10.8%]), and gastric (175 [10.6%]) (Figure). Neoplasia sites with comparatively higher proportions of clinical trials involving surgical interventions were stomach (175 [18.1%]), colorectal (290 [11.7%]), and bladder (60 [11.9%]). Lung and breast cancer had the greatest number of clinical trials, but surgical trials accounted for smaller percentages (125 [4.2%] and 178 [4.9%], respectively).

**Figure.**
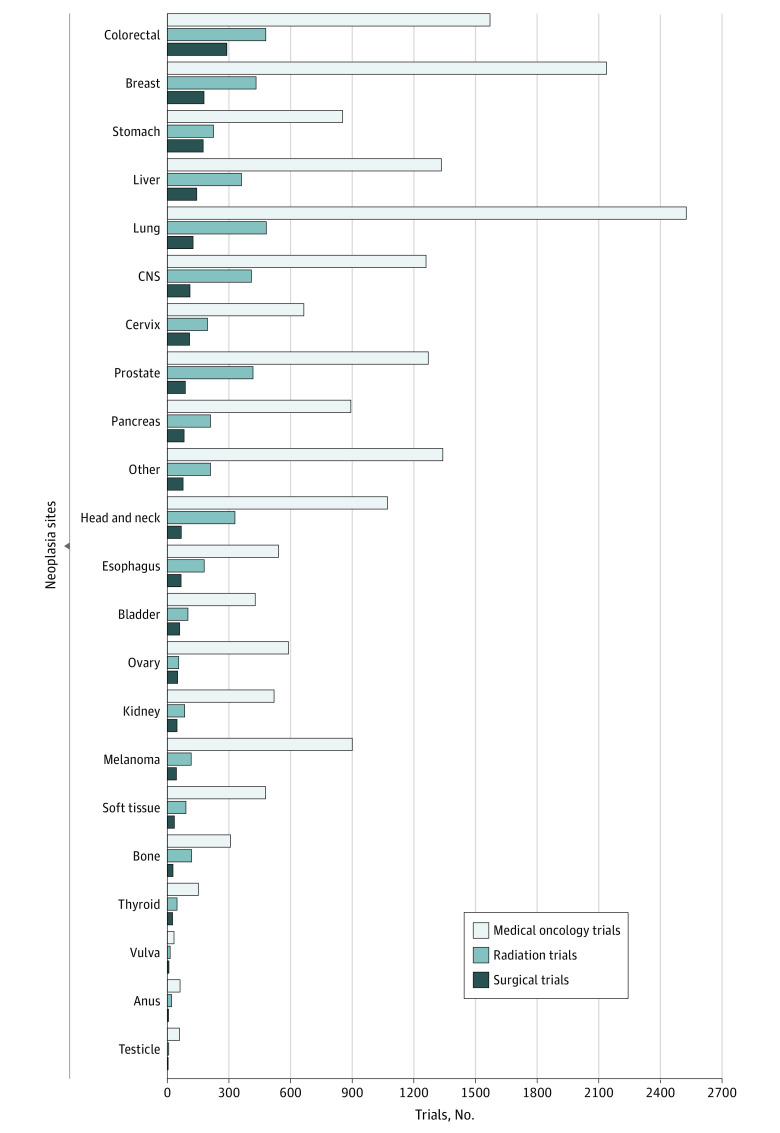
No. of Clinical Trials Involving Surgical, Radiation, and Medical Interventions by Neoplasia Site The number of surgical oncology, radiation oncology, or medical oncology trials are shown for each neoplasm site from highest number of surgical oncology trials to the lowest number of surgical oncology trials.

## Discussion

Although surgical interventions are crucial to treating patients with cancer and determining optimal surgical approaches improves outcomes, there is a paucity of surgical oncology trials. Between 2008 and 2020, only 7.6% of oncology clinical trials investigated surgical interventions, which is lower than previously reported^1^ and reflects low proportions of cancer research involving surgery worldwide.^2^ Study limitations included being restricted to registered trials and dependent on the accuracy of reporting in the ClinicalTrial.gov database.

Studying surgical interventions can be challenging because of limited funding, training, or support for clinical investigators.^5^ Learning from the successes of seminal colorectal and gastric cancer trials, increased surgeon involvement in cooperative research groups (eg, Alliance, NRG Oncology) would advance the field. Development of additional cooperative research groups for understudied neoplasm sites may also increase involvement of more surgical specialties.

With limited surgical oncology trials, trial quality and timely result reporting are crucial. Trials funded by the US government had the lowest hazard of early discontinuation, thus increased support by the National Cancer Institute and other US government funding will address challenges, such as insufficient long-term funding and inadequate accrual of patients, which may occur with industry or academic funding.

Increasingly, surgical clinical trials originate from outside of the US.^6^ Advocacy for US government funds for surgical oncology trials could improve cancer care and allow the US to continue contributing to surgical oncology advances.
